# Influence of genetic polymorphisms on serum biomarkers of cardiac health

**DOI:** 10.1097/MD.0000000000033953

**Published:** 2023-06-09

**Authors:** Hari Krishnan Krishnamurthy, Uma Maheshwari Balaguru, Michelle Pereira, Vasanth Jayaraman, Qi Song, Karthik Krishna, Tianhao Wang, Kang Bei, John J. Rajasekaran

**Affiliations:** a Vibrant Sciences LLC., San Carlos, CA; b Vibrant America LLC., San Carlos, CA.

**Keywords:** cardiac markers, genetic polymorphism, LDL, polygenic risk score, risk assessment

## Abstract

Cardiovascular diseases (CVDs) are a leading cause of death worldwide which is why early risk prediction is crucial. Discrete Polygenic risk score (PRS) measurement using saliva or dried blood spot samples collected at home poses a convenient means for early CVD risk assessment. The present study assessed the effects of 28 disease-associated single nucleotide polymorphisms (SNPs) on 16 serological cardiac markers and also aggregated the risk alleles into a PRS to evaluate its applicability in CVD-risk prediction. The study assessed genetic and serological markers in 184 individuals. The association between serological markers and individual genetic variants was evaluated using a two-tailed *t* test while the associations of serum markers with the PRS was analyzed using the Pearson correlation. The comparative analysis of genotypes revealed statistically significant associations between serum markers and CVD-associated SNPs with Apo B: Apo A-1, LDL Direct, Apo B, sdLDL, hsCRP, Lp(a), NT-proBNP, and PLAC levels being significantly associated with the risk alleles of the SNPs, rs12526453, rs5186, rs10911021, rs1801131, rs670, rs10757274, and rs10757278. Increased PLAC levels were associated with rs10757274 and rs10757278 (*P* < .05). The SNPs, rs1801133, rs1549758, rs1799983, rs5082, and rs5186 were significantly associated with an increase in the cardioprotective markers, HDL and ApoA1 (*P* < .05). Furthermore, the PRS was associated with increasing levels of several serum cardiac markers (*r*^2^ > 0.6). Significant correlations were observed between high PRS and NT-proBNP and ox-LDL levels with the *r*^2^ values being 0.82 (95% CI = 0.13–0.99; *P* = .03) and 0.94 (95% CI = 0.63–0.99; *P* = .005), respectively. The present study reports that SNPs have differential effects on serum markers with rs12526453, rs5186, rs10911021, rs1801131, rs670, rs10757274, and rs10757278 showing significant associations with elevated marker levels, which are indicators of deteriorating cardiac health. Genetic assessment via a convenient at-home collection to calculate the PRS can serve as an effective predictive tool for early CVD-risk assessment. This may help identify the risk groups that may require increased serological monitoring.

## 1. Introduction

Cardiovascular diseases (CVDs) are one of the most common causes of premature and sudden death accounting for approximately 32% of the total deaths worldwide.^[1–4]^ Annual reports from the World Health Organization indicated that despite being among the most preventable causes of death, CVDs are still seen to top the charts for deaths caused by non-communicable diseases.^[5]^ CVDs, which were previously perceived as age-associated conditions have now become exceedingly common among adolescents and young individuals,^[6]^ with the proportion of acute myocardial infarctions having increased from 27% to 32% in patients < 55 years old in the past 20 years.^[7]^ This is believed to stem from the increased prevalence of CVD risk factors such as hypertension, dyslipidemia, diabetes, smoking, obesity, and sedentary lifestyle.^[8]^ As a result, there is a need for early screening tools for CVDs as early risk ascertainment is an important determinant of future events. Oftentimes, heart attacks strike suddenly with no warning, with the earliest symptoms being jaw, neck, or back discomfort, lightheadedness, chest uneasiness, arm or shoulder discomfort, and shortness of breath.^[9]^ These symptoms are immediately followed by a heart attack. This leaves little to no time for treatment, thereby increasing CVD mortality. For this reason, risk assessment strategies based on factors such as demographics, (age and gender), hematological (cholesterol, creatine kinase-myocardial band), and vital parameters (blood pressure) were established to screen for CVDs.^[10,11]^ However, these conventional risk assessment components are subject to change owing to factors such as lifestyle, stress, and other related health conditions or complications. Yildiz et al, 2022, attempted to find more concrete parameters and found that lesion properties (left anterior descending lesion, proximally located lesion), no-reflow resulting in inadequate myocardial perfusion, and prolonged ischemia time were better determinants for the left ventricular ejection fraction decline.^[10]^ Similarly, Uygur et al,^[12]^ demonstrated that epicardial adipose tissue volume could be used in risk stratification for major adverse cardiovascular events. Although concrete, these parameters need to be assessed via sophisticated cardiac imaging techniques which do not make them favorable for mass screening. Alternatively, genetic factors which can be assessed using simple laboratory techniques are seen to strongly correlate with the incidence of CVDs.^[13]^

Many epidemiological studies showed that 40% to 50 % of CVD cases are associated with predisposing genetic risk factors.^[13,14]^ Genetic mutations are present from birth and remain uninfluenced by environmental factors thus, making them attractive candidates for the establishment of early diagnostic strategies. Genome-wide association studies have risen as potential tools for identifying these mutations, especially single nucleotide polymorphisms (SNPs). Various genome-wide association studies targeting the genetics of CVDs have identified millions of functional (located in the coding sequences) and nonfunctional (intergenic) SNPs associated with CVDs.^[15–17]^ Additionally, the convenience associated with the sample collection for genetic testing makes it a favorable tool for early CVD assessment; individuals can collect saliva or dried blood spot samples at home and can send them to a diagnostic laboratory for genetic assessment. The inclusion of genetic testing in risk assessment strategies will aid in the understanding of disease risks, potentially earlier in life, and will enable the creation of tailored risk reduction strategies.^[18]^ However, this inclusion of genetics for CVD risk assessment requires a prior understanding of the underlying mechanism of how these SNPs affect disease progression. Evaluating the association of SNPs with established serum markers may help in elucidating the underlying mechanism, thus helping us understand the influence of SNPs on CVD development. Additionally, for a polygenic disorder like CVD, multiple-risk allele analysis can help in determining disease susceptibility. The aggregation of risk alleles into a polygenic risk score (PRS) can indicate the cumulative risk of genetic variations for a set of traits. Although individual genetic variants confer disease risks, the summation of the risk alleles into a singular score can be superior in disease risk analysis and thus, forms an effective tool for disease prediction.^[18]^

In the present study, we selected 28 SNPs that were previously proven to be associated with CVDs and analyzed their influence on 16 cardiac serological biomarkers. Additionally, based on the risk alleles we calculated the integrated genetic risk via the PRS to understand how the score can help in risk classification and evaluated the score’s associations with serum cardiac marker levels.

## 2. Methods

### 2.1. Study population

The study included 184 subjects who visited Vibrant America for regular or suspected CVD checkups. The study was conducted in a healthy population of nonsmokers (self-reported) who were tested for their cardiac genetic (CardiaX) and serum cardiac biomarker profile. The subjects were all of the European ancestry (self-reported). The data on general variables such as age and sex obtained at the time of sampling were included in the study. This study was done as a retrospective analysis using de-identified laboratory test results under IRB # 1-1098539-1 approved by Western IRB.

### 2.2. Serological markers

A total of 16 serological markers were used in the study. The standard laboratory protocol was followed for the assessment of each marker. Low-density lipoprotein (LDL) (Direct), high-density lipoprotein (HDL), Triglyceride, Lipoprotein-Associated Phospholipase (Lp-PLA2) Test (PLAC), Homocysteine, and small dense low-density lipoprotein-cholesterol (sdLDL) were measured by the enzymatic-colorimetric method using Beckman Coulter AU680 clinical analyzer. Myeloperoxidase (MPO) was measured by latex enhanced Immunoturbidimetric method and total cholesterol was measured by the cholesterol dehydrogenase method using Beckman Coulter AU680 clinical analyzer. Oxidized low-density lipoprotein (ox-LDL) was measured by ELISA using Hamilton Microlab STAR. Apolipoprotein A1 (Apo A-1) and Apolipoprotein B (Apo B) were measured by the immunoturbidometric method, while high-sensitivity C-reactive protein (hs-CRP) and Lipoprotein(a) (Lp(a)) were measured by the particle-enhanced immunoturbidimetric assay using Roche Cobas 6000 c 501. N-terminal pro b-type natriuretic peptide (NT-proBNP) was measured by the electrochemiluminescence Immunoassay using Roche Cobas 6000 c 501. The concentration of LDL cholesterol (denoted as LDL-C) was calculated using triglycerides and HDL concentration using Friedewald’s formula (LDL cholesterol = total cholesterol − HDL cholesterol − (triglycerides/5).

### 2.3. Candidate SNPs and genotyping

28 previously reported CVD risk-associated SNPs were included in the study. The selected SNPs were from multiple gene loci and the functional variants of these genes have been involved in various processes like lipid synthesis, inflammation, vascular homeostasis, and metabolism. Details of the SNPs are given in Supplemental Digital Content 1, http://links.lww.com/MD/J102. For genotyping, genomic DNA was isolated from blood samples and genetic analysis was carried out by the PCR-RFLP method using a Real-Time PCR System.

### 2.4. Polygenic risk score

For calculating the PRS we followed the method described in Samuli Ripatti et al, 2010 and Kathiresan S et al, 2008.^[19,20]^ For each copy of the risk allele, a weight of 1 was applied to a given SNP and the cumulative score was calculated for all 28 SNPs. The maximum cumulative score obtained was 56. Here, 0 indicated no risk alleles and 56 indicated the presence of all risk alleles for the given 28 SNPs.

### 2.5. Statistical analysis

Clinical data were expressed as mean ± standard deviation for normally distributed continuous variables. Intergroup differences in continuous variables were assessed using a two tailed *t* test. Genotype distributions and allele frequencies were calculated by the gene-counting method. The effect of genotypes on serological markers was evaluated using a two-tailed *t* test by performing the analysis separately for each genotype. Univariate Pearson correlation was used to analyze the association of the PRS with serum marker levels. All statistical analyses were carried out using the commercial GraphPad Prism version 9.0 statistics software. A two-tailed *P* value of .05 was considered statistically significant for the study.

## 3. Results

### 3.1. Baseline characteristics and genotype distribution

The present study evaluated the association of 16 cardiac serological biomarkers with 28 CVD-associated SNPs in 184 individuals. The overall study population included 59% men and 41% women subjects with the mean age of men being 50.83 ± 15.39 years while that for women was 47.54 ± 15.5 years. The baseline characteristics of the subjects are given in Table 1. The frequencies of the mutant genotypes for most of the genes ranged between 2% to 59% with an exception of rs138326449, which is a known rare variant where 99% of the subjects were of the wild type. The mutant allele’s expression was considerably higher in comparison to the wild type for the SNPs, rs169713 (mutant type 59% vs wild type 7%), rs12526453 (mutant type 54% vs wild type 8%), and rs10911021 (mutant type 47% vs wild type 9%). Additionally, the mutant allele’s expression was moderately higher than the wild type for the SNPs rs2383207, rs2383206, rs4646994, and rs1799998. The frequency of the heterozygous genotype in the study population ranged from 7% to 52 %. The details of the SNPs used in the study are given in Supplemental Digital Content 1, http://links.lww.com/MD/J102. The frequency of the three genotypes, i.e., homozygous wild, heterozygous and homozygous mutant for all SNPs analyzed in the study are given in Table 2.

**Table 1:** Baseline characteristics of subjects.

Characteristics	Men (n = 110, 59%)	Women (n = 74, 41%)
Mean **a**ge (yr)	50.83 ± 15.39	47.54 ± 15.5
Cholesterol, **total** (mg/dL)	186.66 ± 43.09	193.29 ± 38.16
LDL **d**irect (mg/dL)	125.22 ± 39.7	121.17 ± 33.83
HDL **d**irect(mg/dL)	48.61 ± 13.42	58.67 ± 19.81
Triglyceride (mg/dL)	111.25 ± 61.38	111.05 ± 59.98
Apo A-1 (mg/dL)	153.06 ± 33.06	175.29 ± 41.49
Apo B (mg/dL)	100.74 ± 28.66	99.2 ± 28.02
Lp(a) (mg/dL)	37.05 ± 35.18	34.58 ± 28.59
hs-CRP (mg/dL)	2.28 ± 5.87	3.55 ± 6.93
Homocysteine (µmol/L)	10.34 ± 3.21	8.53 ± 3.4
NT-proBNP (pg/mL)	102.99 ± 251.46	126.86 ± 227.49
sdLDL (mg/dL)	36.16 ± 15.32	31.98 ± 16.06
ox-LDL (U/L)	49.78 ± 20.32	46.14 ± 19.63
MPO (pmol/L)	1087.79 ± 1029.19	987.87 ± 1009.33
Apo B: Apo A-1 (U/L)	0.66 ± 0.21	0.57 ± 0.2
LDL **c**alculation (mg/dL)	116.82 ± 38.69	112.41 ± 30.44
PLAC (ng/mL)	186.08 ± 48.08	147.37 ± 33.83

The values are presented as means ± SD with their respective units.

Apo A-1 = Apolipoprotein A1, Apo B = Apolipoprotein B, HDL = high-density lipoprotein, hs-CRP = high-sensitivity C-reactive protein, LDL = low-density lipoprotein, Lp(a) = Lipoprotein(a), MPO = Myeloperoxidase, NT-proBNP = N-terminal pro b-type natriuretic peptide, Ox-LDL = oxidized low-density lipoprotein, PLAC = Lipoprotein-Associated Phospholipase (Lp-PLA2) Test, sdLDL = small dense low-density lipoprotein-cholesterol.

**Table 2:** Frequencies of genotypic and allelic polymorphisms in the study population.

SNP	Allele	SNP type	Genotype frequency			Allele frequency	
			Major	Heterotype	Minor	Major	Minor
**rs10757274**	A/G	9p21	AA (51, 28%)	AG (93, 51 %)	GG (40, 22%)	0.53	0.47
**rs10757278**	A/G	9p21	AA (51, 28%)	AG (94, 51 %)	GG (39, 21%)	0.53	0.47
**rs2383207**	A/G	9p21	AA (34, 18%)	AG (87, 47 %)	GG (63, 34%)	0.42	0.58
**rs2383206**	A/G	9p21	AA (42, 23%)	AG (93, 51 %)	GG (49, 27%)	0.48	0.52
**rs169713**	C/T	6p24.1	CC (12, 7%)	CT (63, 34 %)	TT (109, 59%)	0.24	0.76
**rs12526453**	C/G	6p24.1	CC (15, 8%)	CT (70, 38%)	TT (99, 54%)	0.27	0.73
**rs2200733**	C/T	4q25	CC (134,73%)	CT (47, 26 %)	TT (3, 2%)	0.86	0.14
**rs10033464**	G/T	4q25	GG (137,74)	GT (41, 22 %)	TT (6, 3%)	0.86	0.14
**rs4680**	A/G	COMT	AA (49, 27%)	AG (92, 50 %)	GG (43, 23%)	0.52	0.48
**rs4646994**	D/I	ACE	DD (40, 22%)	DI (96, 512%)	II (48, 26%)	0.48	0.52
**rs10911021**	C/T	1q25	CC (16, 9%)	CT (81, 44 %)	TT (87, 47%)	0.31	0.69
**rs1801133**	C/T	MTHFR	CC (71, 39%)	CT (90, 49%)	TT (23, 13%)	0.63	0.37
**rs1801131**	C/T	MTHFR	CC (92, 50%)	CT (73, 40%)	TT (19, 10%)	0.7	0.3
**rs2472297**	C/T	15q24.	CC (131,71%)	CT (48, 26 %)	TT (5, 3%)	0.84	0.16
**rs762551**	A/C	CYP1A2	AA (96, 52%)	AC (71, 39%)	CC (17, 9%)	0.71	0.29
**rs4238001**	C/T	SCARB1	CC (151,82%)	CT (33, 18 %)	TT (0, 0%)	0.91	0.09
**rs1799998**	C/T	CYP11B2	CC (29, 16%)	CT (86, 47%)	TT (69, 38%)	0.39	0.61
**rs1050450**	C/T	GSHPx	CC (98, 53%)	CT (71, 39 %)	TT (15, 8%)	0.73	0.27
**rs3918226**	C/T	NOS3	CC (170,92%)	CT (13, 7%)	TT (1, 1%)	0.96	0.04
**rs1549758**	C/T	NOS3	CC (106, 58%)	CT (69, 38%)	TT (9, 5%)	0.76	0.24
**rs1799983**	G/T	NOS3	GG (102,55%)	GT (73, 40 %)	TT (9, 5%)	0.75	0.25
**rs1042714**	C/G	ADR-B2	CC (82, 45%)	CG (76, 41 %)	GG (26, 14%)	0.65	0.35
**rs670**	A/G	APOA1/C3/A5	AA (118,64%)	AG (62, 34 %)	GG (4, 2%)	0.81	0.19
**rs5082**	C/T	ApoA2	CC (87, 47%)	CT (84, 46 %)	TT (13, 7%)	0.7	0.3
**rs1126742**	C/T	CYP4A11	CC (162,88%)	CT (20, 11 %)	TT (2, 1%)	0.07	0.93
**rs2108622**	C/T	CYP4F2	CC (97, 53%)	CT (73, 40%)	TT (14, 8%)	0.73	0.27
**rs5186**	A/C	AGTR1	AA (105, 57%)	AC (66, 36%)	CC (13, 7%)	0.75	0.25
**rs138326449**	A/G	ApoC3	AA (1, 1%)	AG (0, 0%)	GG (183,99%)	0.01	0.99

### 3.2. Effect of individual polymorphisms on cardiovascular risk marker levels

In order to establish the association between SNPs and serum cardiac markers, the differences in the serological marker levels were compared across all genotypes. The average levels of the respective serum cardiac markers for all three genotypes in association with the 28 SNPs are given in Supplemental Digital Content 2, http://links.lww.com/MD/J103. The differences between the wild vs mutant and wild vs heterozygous genotypes were assessed for significance using a two-tailed *t* test. The markers that showed significant differences (*P* < .05) between genotypes are given in Tables 3 and 4.

**Table 3:** Association between serum cardiac markers and single nucleotide polymorphisms observed in subjects with wild and mutant genotypes.

SNP	Marker	Wild type	Mutant type	Difference	Effect on levels	*P* value (W vs M)
rs10757274	PLAC	156.95	171.90	14.95	High	.021
rs10757278	PLAC	156.48	169.00	12.52	High	.023
rs12526453	LDL direct	106.40	128.60	22.20	High	.036
	Apo B	85.69	101.49	15.80	High	.022
	Homocysteine	12.79	9.61	3.18	Low	.040
	sdLDL	29.32	33.96	4.64	High	.035
	ox-LDL	39.37	49.48	10.12	High	.025
	Apo B: Apo A-1	0.53	0.65	0.12	High	.028
rs4646994	NT-proBNP	76.32	55.77	20.55	Low	.047
rs1801133	HDL direct	51.32	59.00	7.68	High	.014
	Apo A-1	157.32	177.88	20.56	High	.036
rs1801131	PLAC	168.19	54.77	13.42	Low	.028
rs1799998	HDL direct	60.37	48.91	11.46	Low	.002
	Apo A-1	178.25	156.51	21.74	Low	.009
rs1050450	Lp(a)	39.58	16.67	22.91	Low	.034
	Apo B: Apo A-1	0.63	0.46	0.17	Low	.050
rs1549758	HDL direct	53.93	61.00	7.07	High	.018
	Apo A-1	165.13	182.12	16.99	High	.016
	ox-LDL	47.37	40.51	6.86	Low	.044
	Apo B: Apo A-1	0.65	0.55	0.10	Low	.038
rs1799983	HDL direct	53.09	63.43	10.34	High	.039
	Apo A-1	162.87	189.37	26.50	High	.028
rs670	NT-proBNP	103.10	92.19	10.91	Low	.000
	MPO	1068.06	433.02	635.04	Low	.005
rs2108622	Apo B: Apo A-1	0.60	0.75	0.16	High	.036
rs5186	sdLDL	33.71	37.70	3.99	High	.007

Apo A-1 = Apolipoprotein A1, Apo B = Apolipoprotein B, HDL = high-density lipoprotein, LDL = low-density lipoprotein, Lp(a) = Lipoprotein(a), MPO = Myeloperoxidase, NT-proBNP = N-terminal pro b-type natriuretic peptide, Ox-LDL = oxidized low-density lipoprotein, PLAC = Lipoprotein-Associated Phospholipase (Lp-PLA2) Test, sdLDL = small dense low-density lipoprotein-cholesterol.

**Table 4:** Association between serum cardiac markers and single nucleotide polymorphisms observed in subjects with wild and hetero or carrier allele genotype.

SNP	Marker	Wild type	Heterozygous type	Difference	Effect on levels	*P* value (W vs H)
**rs12526453**	Homocysteine	12.79	9.34	3.45	Low	.037
**rs10911021**	Lp(a)	26.89	18.02	10.46	High	.027
	hs-CRP	2.90	4.38	1.48	High	.028
**rs1801131**	Lp(a)	39.81	31.59	8.22	High	.041
**rs1050450**	Ox LDL	52.094	43.634	8.46	Low	.010
**rs1549758**	ox-LDL	47.37	48.11	0.74	High	.054
**rs670**	Cholesterol, Total	192.64	178.65	13.99	Low	.042
	hs-CRP	2.63	4.07	1.44	High	.014
**rs5082**	Apo A-1	163.26	169.23	5.97	High	.036
	HDL direct	52.14	56.86	4.72	High	.012
**rs5186**	Apo A-1	161.41	170.88	9.47	High	.037
	HDL direct	52.48	57.152	4.672	High	.040

Apo A-1 = Apolipoprotein A1, HDL = high-density lipoprotein, hs-CRP = high-sensitivity C-reactive protein, Lp(a) = Lipoprotein(a), Ox-LDL = oxidized low-density lipoprotein.

On comparing the wild and mutant genotypes, 13 SNPs were significantly associated with altered serum cardiac marker levels, with 7 SNPs affecting two or more markers (Table 3). The polymorphism, rs12526453 was significantly associated with high LDL Direct (*P* = .036) and high Apo B (*P* = .022) levels which are both risk factors for CVD. Apo B: Apo A-1 ratio was significantly increased in correlation with the mutant genotypes of rs12526453 (*P* = .028) and rs2108622 (*P* = .036) while it was decreased for rs1050450 (*P* = .050) and rs1549758 (*P* = .038), which can be considered protective against CVD. sdLDL levels were increased in association with rs12526453 (*P* = .035) and rs5186 (*P* = .007). The SNP, rs12526453 (*P* = .025) increased the risk marker, ox-LDL, while rs1549758 (*P* = .044) reduced ox-LDL levels. Similarly, rs10757274 (*P* = .021) and rs10757278 (*P* = .023) were seen to increase serum PLAC levels while rs1801131 (*P* = .028) was associated with reduced PLAC levels. rs1799998 was seen to reduce HDL Direct (*P* = .002) and Apo A-1 levels (*P* = .009) (Table 3).

Several SNPs exerted protective effects against CVD by reducing the levels of the risk markers including NT-proBNP, Homocysteine, Lp(a), and MPO. rs4646994 (*P* = .047) and rs670 (*P* = .000) were associated with lower NT-proBNP levels, while rs12526453 and rs1050450 were associated with lower Homocysteine (*P* = .040) and Lp(a) (*P* = .034) levels respectively. Additionally, rs670 also reduced MPO levels (*P* = .005). The SNPs, rs1801133, rs1549758, and rs1799983 had increased HDL Direct and Apo A-1 levels, which are protective against CVD (*P* < .05) (Table 3). The serological marker-wise effect of these polymorphisms has been summarized in Table 5.

**Table 5:** Serum cardiac marker-wise effect of single nucleotide polymorphisms observed in subjects with wild and mutant genotypes.

Marker	SNP	Effect on levels
LDL direct	rs12526453	High
Apo B	rs12526453	High
Apo B: Apo A-1	rs12526453, rs2108622	High
	rs1050450, rs1549758	Low
sdLDL	rs12526453, rs5186	High
ox-LDL	rs12526453	High
	rs1549758	Low
PLAC	rs10757274, rs10757278	High
	rs1801131	Low
HDL direct	rs1801133, rs1549758, rs1799983	High
	rs1799998	Low
NT-proBNP	rs4646994, rs670	Low
Homocysteine	rs12526453	Low
Lp(a)	rs1050450	Low
MPO	rs670	Low
Apo A-1	rs1801133, rs1549758, rs1799983	High
	rs1799998	Low

Apo A-1 = Apolipoprotein A1, Apo B = Apolipoprotein B, HDL = high-density lipoprotein, LDL = low-density lipoprotein, Lp(a) = Lipoprotein(a), MPO = Myeloperoxidase, NT-proBNP = N-terminal pro b-type natriuretic peptide, Ox-LDL = oxidized low-density lipoprotein, PLAC = Lipoprotein-Associated Phospholipase (Lp-PLA2) Test, sdLDL = small dense low-density lipoprotein-cholesterol.

On comparison between wild and heterozygous genotypes, 8 SNPs were found to be significantly associated with altered serum cardiac marker levels (Table 4). Ox LDL levels were high for rs1549758 (*P* = .054) while they were low for rs1050450 (*P* = .010). The SNPs, rs10911021 (*P* = .027) and rs1801131 (*P* = .041) had high Lp(a) levels. High-sensitivity C-reactive protein levels were associated with the polymorphisms, rs10911021 (*P* = .028) and rs670 (*P* = .014). rs12526453 had low Homocysteine levels (*P* = .037) while rs670 had low Cholesterol levels (*P* = .042), which are both risk markers for CVD. Thus, low levels of these markers are beneficial to cardiac health. Lastly, the SNPs, rs5082 and rs5186 seemed to have cardioprotective effects as they had high levels of the protective markers, Apo A-1 and HDL Direct (*P* < .05). The serological marker-wise effect of these polymorphisms has been summarized in Table 6.

**Table 6:** Serum cardiac marker-wise effect of single nucleotide polymorphisms observed in subjects with wild and heterozygous genotypes.

Marker	SNP	Effect on levels
Ox LDL	rs1549758	High
	rs1050450	Low
Lp(a)	rs10911021, rs1801131	High
hs-CRP	rs10911021, rs670	High
Homocysteine	rs12526453	Low
Cholesterol, total	rs670	Low
Apo A-1	rs5082, rs5186	High
HDL direct	rs5082, rs5186	High

Apo A-1 = Apolipoprotein A1, HDL = high-density lipoprotein, hs-CRP = high-sensitivity C-reactive protein, Lp(a) = Lipoprotein(a), Ox-LDL = oxidized low-density lipoprotein.

### 3.3. Effect of polygenic risk score on cardiovascular risk marker levels

**Figure 1.**
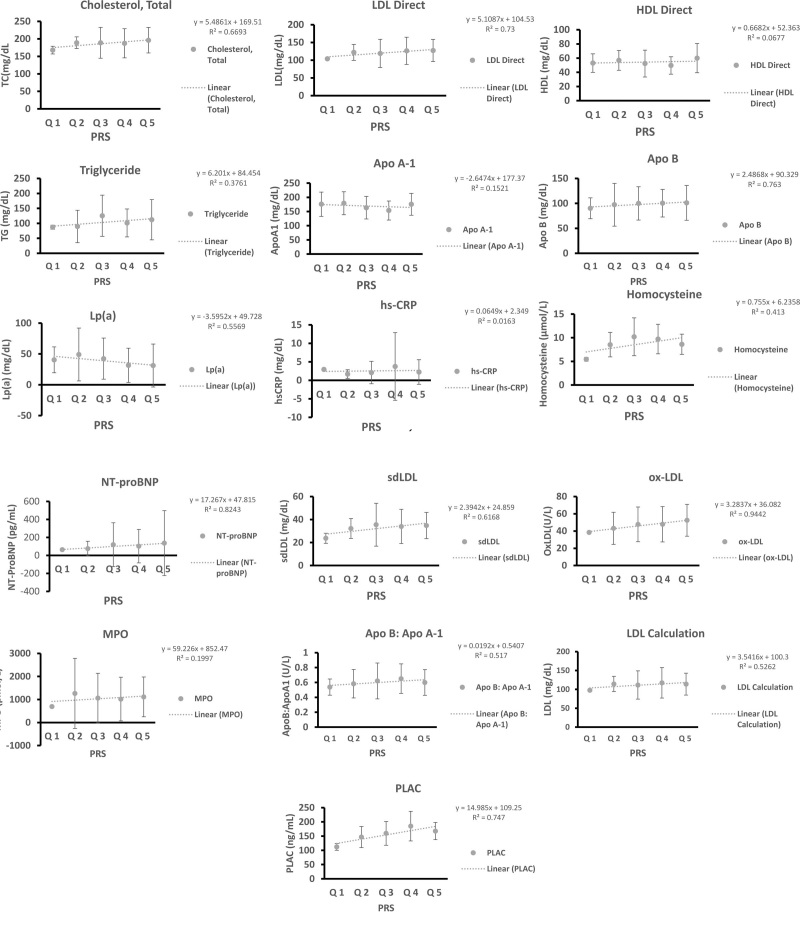
Shows the effect of the PRS on the following serum markers: A-total cholesterol, B-LDL direct, C-HDL direct, E-Triglyceride, F-ApoA1, G- ApoB, H-Lp(a), I-hsCRP, J-Homocysteine, K-NTproBNP, L-sdLDL, M-oxLDL, N-MPO, O-ApoB: ApoA1, P-LDL calculation, and Q-PLAC. The PRS was calculated as sum of the risk alleles possessed by each individual participant. Participants were stratified into Quintile 1 consisting of PRS scores between 1 to 10 (n = 2), Quintile 2 consisting of PRS scores between 10 to 15 (n = 8), Quintile 3 consisting of PRS scores between 15 to 20 (n = 69), Quintile 4 consisting of PRS scores between 20 to 25 (n = 76) and Quintile 5 consisting of PRS scores between 25 to 30 (n = 29). Apo A-1 = Apolipoprotein A1, Apo B = Apolipoprotein B, HDL = high-density lipoprotein, hs-CRP = high-sensitivity C-reactive protein, LDL = low-density lipoprotein, Lp(a) = Lipoprotein(a), PLAC = Lipoprotein-Associated Phospholipase (Lp-PLA2) Test, MPO = myeloperoxidase, PRS = polygenic risk score, sdLDL = small dense low-density lipoprotein-cholesterol.

Early detection is key in CVD risk mitigation and comprehensive disease-specific SNPs that have been validated across several studies pose as a good tool for risk assessment. A PRS-based system which integrates these variants can be used to classify individuals from low to high risk based on the aggregate number of risk alleles carried by the individual. As we observed significant associations between SNPs and multiple serum markers, we calculated the PRS for the 28 SNPs in an attempt to evaluate the association of the PRS with the 16 serological markers. Increased levels of the two serum markers, NT-proBNP and ox-LDL were statistically significant with increasing PRS with their *r* values being 0.82 (95% CI = 0.13–0.99; *P* = .03) and 0.94 (95% CI = 0.63–0.99; *P* = .005) respectively (Table 7; Fig. 1). Although significance was not obtained for the other serum cardiac markers, the results did indicate that an increase in marker levels was associated with increasing PRS (Table 7; Fig. 1).

**Table 7:** Correlation between the polygenic risk score and serum cardiac markers.

Serum marker	*R* squared value	95% confidence interval	*P* (two-tailed)
Total **cholesterol**	0.6693	(−0.23 to 0.99)	.091
LDL **direct**	0.73	(−0.11 to 0.99)	.065
HDL **direct**	0.0677	(−0.80 to 0.93)	.673
Triglyceride	0.3761	(−0.58 to 0.97)	.271
Apo A-1	0.1521	(−0.95 to 0.75)	.516
Apo B	0.763	(−0.038 to 0.99)	.053
Lp(a)	0.5569	(−0.98 to 0.40)	.147
hs-CRP	0.0163	(−0.85 to 0.91)	.838
Homocysteine	0.413	(−0.55 to 0.97)	.242
NT-proBNP	0.8243	(0.13–0.99)	.033*
sdLDL	0.6168	(−0.31 to 0.98)	.115
ox-LDL	0.9442	(0.63–0.99)	.005*
MPO	0.1997	(−0.71 to 0.95)	.451
Apo B: Apo A-1	0.517	(−0.44 to 0.98)	.171
LDL calculation	0.5262	(−0.44 to 0.98)	.165
PLAC	0.747	(−0.076 to 0.99)	.059

*P < 0.05.

Apo A-1 = Apolipoprotein A1, Apo B = Apolipoprotein B, HDL = high-density lipoprotein, hs-CRP = high-sensitivity C-reactive protein, LDL = low-density lipoprotein, Lp(a) = Lipoprotein(a), MPO = Myeloperoxidase, NT-proBNP = N-terminal pro b-type natriuretic peptide, Ox-LDL = oxidized low-density lipoprotein, PLAC = Lipoprotein-Associated Phospholipase (Lp-PLA2) Test, sdLDL = small dense low-density lipoprotein-cholesterol.

## 4. Discussion

CVDs are a major public health concern. They are often silent or may strike without warning, which highlights the need for early CVD risk prediction. Although traditional markers such as LDL, HDL, and cholesterol levels have been effective in screening and diagnosing CVDs, they are modifiable by lifestyle factors and are hence questionable in context of long-term CVD predictability.^[21]^ As opposed to traditional markers, genetic markers have risen as attractive candidates for CVD risk assessment owing to their independent associations with CVD outcomes. The strength of genetic markers in risk analysis is that they are present from birth and are not influenced by environmental factors.^[22]^ Genetic testing has risen as an attractive tool for CVD risk prediction and mitigation.^[18]^ Additionally, studies have shown that for polygenic diseases like CVD, the aggregation of risk alleles into a PRS forms a far superior tool in risk assessment in comparison to individual genetic variation for a set of traits.^[18]^

The present study evaluated the association of 28 CVD SNPs with 16 cardiac serological markers and also assessed the influence of the PRS on serum cardiac marker levels. The comparative analysis of genotypes revealed several statistically significant associations between serum markers and CVD-associated SNPs. The increased levels of the markers, Apo B: Apo A-1, LDL Direct, Apo B, sdLDL, hsCRP, Lp(a), NT-proBNP, and PLAC levels were found to be significantly associated with the risk allele carriers of the SNPs, rs12526453, rs5186, rs10911021, rs1801131, rs670, rs10757274, and rs10757278 (*P* < .05). The polymorphism, rs12526453 is heavily implicated in the risk of CVD as its mutant genotype was significantly associated with increased levels of LDL, sdLDL, oxLDL, Apo B: Apo A-1, and ApoB (*P* < .05). These observations are in line with the results discussed by Han Xia et al 2014, where the phosphatase-related single nucleotide polymorphism, rs12526453 was significantly associated with coronary artery disease (CAD) risk in multiple populations.^[23]^ rs10757274 and rs10757278 were seen to increase the levels of the risk marker, PLAC (*P* < .05), thus, these polymorphisms can be considered as major risk factors contributing to the risk of developing CVDs as described earlier.^[24,25]^ Nevertheless, the comparative analysis also revealed that the SNPs, rs1801133, rs1549758, rs1799983, rs5082, and rs5186 were found to be significantly associated with cardioprotective markers such as HDL and ApoA1. Apolipoprotein A (Apo A1) is a protein carried in HDL and they together help in reducing the levels of cholesterol in the bloodstream. Thus, elevated HDL and ApoA1 are associated with increased cholesterol elimination which is beneficial for cardiac health.^[26]^ While the homozygous risk allele carriers of rs670 were significantly associated with reduced NT-proBNP and MPO levels, the heterozygous genotype for the SNP was associated with high-sensitivity C-reactive protein levels (*P* < .05). Although the correlations were modest and elicited non-uniform trends of association, these correlations can have profound effects on an individual’s health and should be studied further to understand their effect on CVD outcomes.

Following the SNPs and serum marker association, we attempted to establish a PRS system for the 28 SNPs included in the study and evaluate the PRS’s association with serum cardiac markers. We found that the increase in NT-proBNP and ox LDL marker levels were associated with increasing PRS (*P* = .03 and *P* = .005 respectively). Previous studies have also demonstrated strong associations between PRS and CAD. In a study by Natarajan et al, 2017, where a PRS was established to determine the risk of atherosclerosis, the PRS not only aided in risk stratification but also helped in identifying high-risk individuals that were more likely to benefit from primary prevention via statin therapy.^[27]^ We also found that rs169713, rs2383207, rs2383206, rs12526453, rs4646994, rs10911021, and rs1799998, regardless of their high frequency were associated with more than 6 markers and when analyzed separately were found to majorly contribute to the weight of the PRS. Thus, these SNPs appear to be promising SNPs for future studies and prospective targets for cardiac genetic panels. It was also observed that heterozygous (Table 4) individuals (single copy risk allele carriers) of the polymorphisms, rs169713, rs2383207, rs2383206, rs4646994, and rs1799998 contributed to the weight of PRS. This supports previous observations of heterozygous carriers for rare mutations having a several-fold increase in the risk for various common diseases.^[28]^ Future studies should give importance to the heterozygous SNPs that contribute to the PRS and must investigate their role in leading to the increase in risk marker levels.

CVD risks are modifiable and can be reduced by improving diet and lifestyle practices. Previous studies have reported that individuals with good cardiovascular health practices are associated with a reduced risk of CVD incidence and mortality.^[29]^ A study by Khera et al^[30]^, 2016, which evaluated 50 CAD-associated SNPs showed that individuals with high polygenic risk who followed a healthy lifestyle (no smoking, no obesity, physical activity at least once weekly, and consumption of a healthy-diet) had 50% decreased coronary event rates than those individuals with unfavorable lifestyles. In order to maintain good cardiovascular health, the American Heart Association has put together seven healthy practices that will promote cardiovascular health and ultimately reduce the overall burden of CVDs. These factors include no smoking, maintaining an ideal body mass index, exercising regularly, consuming a healthy diet, and maintaining an optimal profile of serum cholesterol, blood pressure, and blood glucose levels, along with other markers that are indicative of good cardiovascular health.^[31]^

Early diagnosis is key in preventing and improving the risks associated with CVD. Autopsy studies have indicated that atherosclerosis begins in early in life with the presence of aortic fatty streaks in children, as early as 10 years of age.^[32]^ This indicates that assessment of cardiovascular health may need to be initiated earlier on to allow early intervention to decrease the atherosclerotic process. Research could focus on understanding the appropriate age at which routine cardiac assessments must be initiated for early prevention of CVD risks. The presence of genetic markers from birth and their ability to remain uninfluenced throughout life makes genetic testing an appropriate tool for early CVD risk prediction. Apart from its predictive effectiveness, the feasibility of genetic testing for early risk assessment makes it an attractive prospect for CVD screening as it can be done from the comfort of one’s home. Saliva or dried blood spot kits are available for easy collection and extraction of DNA for genetic assessment by clinical laboratories. The rising convenience related to the accessibility and feasibility of genetic testing via test kits might help people get familiarized with the test kits as early tools for risk assessment. This may prospectively lead to increased early CVD risk assessment, thereby reducing the burden of CVDs.

In this study, we found that CVD polymorphisms have differential effects on serum markers which are indicative of poor cardiac health. In such scenarios, individuals at high risk must be monitored and treated with robust interventional and medical approaches as needed. These individuals must adopt healthy dietary and lifestyle practices which are supportive of heart health. Additionally, high risk individuals must routinely monitor their cardiac biomarker profile via regular serological testing. Regular monitoring will also help in understanding the success of the given interventions/medications. While genetic markers are effective tools for predictive risk assessment, serological markers are actual representations of the body’s status. Hence, both, genetic and serological assessments are important aspects of CVD risk classification and should be a part of CVD risk assessment in clinical settings.

Our study presents an enhanced CVD screening measure by integrating both, genetic and serological risk factors. However, there are a few limitations associated with the study. The study population lacked ethnic diversity. Future studies must aim at assessing SNPs in populations that are free from ethnic limitations. Although the adequacy of our sample size was determined by comparing it with similar studies in the field and performing power analysis, the results from this study must be validated in larger clinical trials. Additionally, as this was a one-time study, it lacks follow-up data that could help support the association of the PRS with increasing risk marker levels and CVD outcomes. Additional analysis in larger confirmed CVD sample sets should be carried out to provide robust validation of the PRS as an effective tool in early risk assessment.

## 5. Conclusion

Early genetic testing can be an effective means of assessing the risks of cardiovascular diseases. Polygenic risk scores that are calculated based on genetic assessment can be performed via a simple at-home collection using saliva or dried blood spot samples. This enables early identification and adoption of preventive measures that promote cardiovascular health. Moreover, high-risk patients can regularly monitor their cardiac health via serological testing to assess the effectiveness of interventions. Our study investigated the implication of the PRS in risk prediction and emphasizes its applicability in clinical settings. PRS prediction along with regular diagnostic testing can help reduce cardiovascular disease burden.

## Acknowledgments

We acknowledge Vibrant America LLC for supporting this research.

## Author contributions

**Conceptualization:** Hari Krishnan Krishnamurthy, Vasanth Jayaraman, John J. Rajasekaran.

**Data curation:** Uma Maheshwari Balaguru, Qi Song, Kang Bei, Hari Krishnan Krishnamurthy.

**Formal analysis:** Qi Song, Kang Bei, Hari Krishnan Krishnamurthy, Uma Maheshwari Balaguru.

**Investigation:** Uma Maheshwari Balaguru, Karthik Krishna, Tianhao Wang, Hari Krishnan Krishnamurthy.

**Methodology:** Hari Krishnan Krishnamurthy, Karthik Krishna, Tianhao Wang.

**Supervision:** Hari Krishnan Krishnamurthy.

**Visualization:** Vasanth Jayaraman, John J. Rajasekaran, Hari Krishnan Krishnamurthy.

**Writing – review & editing:** Hari Krishnan Krishnamurthy, Michelle Pereira, Uma Maheshwari Balaguru.

## Correction

This article was originally published with incomplete information for affiliation b. Affiliation b has been updated from “Vibrant LLC., San Carlos, CA” to “Vibrant America LLC., San Carlos, CA.” In addition, author degrees, abbreviations of keywords, and the author contributions have been updated. Figure 1 has also been re-formatted so that it appears as one image.

## Supplementary Material
