# Supplementary material for: Influence of genetic polymorphisms on serum biomarkers of cardiac health
Source: Medicine (Baltimore). 2023 Jun 9;102(23):e33953. doi: 10.1097/MD.0000000000033953 (PMC10256409; doi:10.1097/MD.0000000000033953)
Supplement: Supplementary file 1 [file medi-102-e33953-s001.pdf]

Influence of genetic polymorphisms on serum biomarkers of cardiac health

Hari Krishnan Krishnamurthy[1*], Uma Maheshwari Balaguru[2], Michelle Pereira[2] Vasanth Jayaraman[1], Qi Song[2] Karthik Krishna[1], Tianhao Wang[1], Kang Bei[1], John J. Rajasekaran[1]

1 Vibrant Sciences LLC., San Carlos, CA, United States of America, 2 Vibrant America LLC., San Carlos, CA, United States of America

Supplementary Digital Content 1. Table that gives the details of the SNPs included in the study

SNP	Allele	Chromosomal location	Gene	Protein	Mutation type	Physiological role
rs10757274	A/G	9p21	CDKN2B-AS1	Cyclin-dependent kinase 4 inhibitor B	Functional mutation in lncRNA	Vessel physiology
rs10757278	A/G	9p21	CDKN2A,CDKN2B	Cyclin-dependent kinase 4 inhibitor B	Functional mutation in lncRNA	Vessel physiology
rs2383207	A/G	9p21	CDKN2B-AS1	Cyclin-dependent kinase 4 inhibitor B	Functional mutation in lncRNA	Vessel physiology
rs2383206	A/G	9p21	CDKN2B-AS1	Cyclin-dependent kinase 4 inhibitor B	Functional mutation in lncRNA	Vessel physiology
rs169713	C/T	6p24.1	near HIVEP1	Zinc finger protein 40	Possibly a functional mutation in the HIVEP1 promoter	Inflammation
rs12526453	C/G	6p24.1	PHACTR1	Phosphatase and actin regulator 1	Functional mutation in intronic region	Vessel physiology
rs2200733	C/T	4q25	PITX2,ENPEP	Paired Like Homeodomain 2	Functional mutation-Intergenic	Cardiac development
rs10033464	G/T	4q25	PITX2,ENPEP	Paired Like Homeodomain 2 and enzyme glutamylaminopeptidase A (GAPA)	Functional mutation-Intergenic	Cardiac development and Reninangiotensin-aldosterone system (RAAS)
rs4680	158G > A	ch 22	COMT, MIR4761	catechol-O-methyltransferase, MicroRNA 4761	Functional mutation-Intergenic, Functional mutation-miRNA class	Metabolism
rs4646994	D/I	ch17	ACE	Angiotensin-converting-enzyme	Insertion (I allele) deletion (D allele) polymorphism	Reninangiotensin-aldosterone system (RAAS)
rs10911021	C/T	1q25	GLUL	Glutamate-amonia ligase	Functional mutation-intergenic	Oxidative stress
rs7412/rs429358	526C>T 388T>C	Ch 19	APOE	Apolipoprotein E	Functional mutation-intergenic	Lipid transport
rs1801133	677C>T	1p36	MTHFR	5,10-methylenetetrahydrofolate reductase	Functional mutation-intronic	Metabolism
rs1801131	1298 A>C	ch1	MTHFR	5,10-methylenetetrahydrofolate reductase	Functional mutation-intronic	Metabolism
rs2472297	C/T	ch 15q24.	CYP1A1/CYP1A2	cytochrome P450 1A1 and 1A2	Functional mutation-intronic	Metabolism
rs762551	A/C	ch 15	CYP1A2	cytochrome P450 1A2	Functional mutation-intronic	Metabolism
rs4238001	C/T	ch 12	SCARB1	Lipoprotein Receptor	Functional mutation-intronic	Lipid transport

rs1799998	C/T	ch 8	CYP11B2	Cytochrome 11B2 aldosterone synthase	Functional mutation-intronic	Reninangiotensin -aldosterone system (RAAS)
rs1050450	198C > T	3p21.3	GPX1	glutathione peroxidase 1	Functional mutation-intronic	Oxidative stress/Inflammation
rs3918226	C/T	ch 7	NOS3	Endothelial nitric oxide synthase	Functional mutation-intronic	Vessel physiology
rs1549758	774C>T	ch 7	NOS3	Endothelial nitric oxide synthase	Functional mutation-intronic	Vessel physiology
rs1799983	894G>T	ch 7	NOS3	Endothelial nitric oxide synthase	Functional mutation-intronic	Vessel physiology
rs1042714	codon 27 G>A	ch 5	ADRB2 gene	β2-adrenergic receptor	Functional mutation-intronic	Inflammation
rs670	-75G/A	ch11	APOA1, APOA1-AS	Apoprotein subtype 1	Functional mutation-intronic	Lipid transport
rs5082	-256T/C	ch 1	APOA2	Apolipoprotein A-II .	Functional mutation-intronic	Lipid transport
rs1126742	C/T	ch 1	CYP4A11	Cytochrome P450	Functional mutation-intronic	Metabolism
rs2108622	C/T	ch 19	CYP4F2	Cytochrome P450 Family 4 Subfamily F Member 2	Functional mutation-intronic	Metabolism
rs5186	A/C	ch 3	AGTR1	Angtiotensin receptor type 1	Functional mutation-intronic	Reninangiotensin -aldosterone system (RAAS)
rs138326449	A/G	ch 11	APOC3	Apolipoprotein C3)	Functional mutation-intronic	Lipid transport
